# Efficacy and Tolerability of Ashwagandha Root Extract in the Elderly for Improvement of General Well-being and Sleep: A Prospective, Randomized, Double-blind, Placebo-controlled Study

**DOI:** 10.7759/cureus.7083

**Published:** 2020-02-23

**Authors:** Sunil B Kelgane, Jaysing Salve, Prasanthi Sampara, Khokan Debnath

**Affiliations:** 1 Emergency Medical Services, Maharashtra Emergency Medical Services, Pune, IND; 2 Internal Medicine, Risk Care Hospital, Thane, IND; 3 SMART Research, Sunshine Hospitals, Hyderabad, IND; 4 Family Medicine, Prakruti Hospital, Mumbai, IND

**Keywords:** ashwagandha, elderly, aging, sleep quality, quality of life, mental alertness

## Abstract

Background

Ashwagandha is an excellent adaptogen that is being used since ancient times in Ayurvedic medicine. Traditionally, it is used for various ailments and general well-being, including the treatment of geriatric patients. Managing quality of life (QoL) remains a challenge for the elderly population, especially joint pain management, sleep, and general well-being. With a growing global elderly population, QoL management with efficient medication and supplementation is the major healthcare requirement.

Objective

The objective of this study was to assess the safety, efficacy, and tolerability of Ashwagandha (*Withania somnifera* (L.) Dunal.) root extract on the improvement of general health and sleep in elderly people.

Methods

This 12-week, prospective, randomized, double-blind, placebo-controlled study was conducted on individuals of either gender aged between 65-80 years. Participants were randomized to receive Ashwagandha root extract at a dose of 600 mg/day (n = 25) orally, or identical placebo capsules with the same dose (n = 25) for 12 weeks. Efficacy was assessed using the WHOQOL-BREF questionnaire, sleep quality, mental alertness on rising, and Physician’s Global Assessment of Efficacy to Therapy (PGAET). The safety and tolerability were assessed using the clinical adverse events reporting and Patient's Global Assessment of Tolerability to Therapy (PGATT).

Results

Statistically significant (P<0.0001) improvement was observed in the Ashwagandha treatment group compared to the placebo. The mean (SD) total score of WHOQOL-BREF improved from 140.53 (8.25) at the baseline to 161.84(9.32) at the end of the study. The individual domain scores were also improved. At baseline, the sleep quality and the mental alertness on rising were comparatively low in both the groups. However, upon intervention, a significant increase in the quality of sleep (P<0.0001) and mental alertness (P<0.034) was observed in the Ashwagandha treatment group when compared to the placebo group. Overall improvement was observed for the general wellbeing, sleep quality, and mental alertness in the study population. The experimental group population displayed good tolerability to the test product and it was reported as safe and beneficial by the study participants.

Conclusion

The study outcomes suggest that Ashwagandha root extract was efficient in improving the QoL, sleep quality, and mental alertness as self-assessed by the elderly participants. The recommended dose used in this study could be effective for the elderly population.

## Introduction

Ayurveda served humanity for thousands of years with better health benefits for various diseases and also for general well-being and rejuvenation. Therefore, Ayurvedic therapy could be an important alternative for the aged population. Combating health-related issues and increasing the quality of life (QoL) including mobility impairment, fatigue, and other conditions is possible through proper Ayurvedic ingredient supplementation.

Ashwagandha, scientifically known as *Withania somnifera* (L.) Dunal., commonly known as Indian Ginseng or Winter cherry is one of the most revered herbs of Ayurveda. Over centuries, this herb has been used as a “Rasayana” or rejuvenator and aided in promoting health and longevity, slowing the aging process, and acted as a revitalizer. Ashwagandha is also known as “Sattvic Kapha Rasayana” for its tremendous impact on growth, body weight, lubricating factor, and major tissue development activities [1]. Many pharmacological studies have been conducted to investigate the multiple medicinal properties of Ashwagandha [2]. The roots of Ashwagandha are mostly used for therapeutic purposes in both Ayurveda and Unani medicine [3]. Studies on rats demonstrated that oral administration of Ashwagandha root extract induces sleep [4]. Scientific exploration proved that Ashwagandha is well tolerated in humans and can boost up the testosterone level while decreasing the cortisol level [5-6]. A recent study on *C. elegans* successfully demonstrated the enhanced longevity of the worm using Ashwagandha root extract [7]. Another study used Ashwagandha root extract on HeLa cells and showed increased telomerase activity, representing an anti-aging effect [8].

Improvement of quality of life (QoL) remains the major hurdle for the aged population due to insomnia. Sleep quality has been considered as an excellent marker for the physical and mental health of the aged population [9]. Cognitive issues can emerge due to obstructive sleep apnea [10] and poor sleep quality [11]. Moreover, poor sleep quality is related to several other medical conditions such as pain sensitivity, depression, kidney function, and other life-threatening health issues [12-13]. The relation of insomnia and mortality in elderly populations in a large cohort has been reported [14].

Aging is a natural phenomenon and an unavoidable physiological, psychological, biochemical condition for humans. The human being is seeking the fountain of youth that can either stop or at least stall the process of aging. Recently, the scientific exploration of the aging process has been seriously considered. Apoptosis and senescence have been studied meticulously in all the model organisms, primates, and humans. The improvement in the healthcare services helped in an increment of the aged population and reduced the mortality rate compared to the last century. According to the projection of the World Population Prospects 2019 data, the present aged population is 9% in 2019 which could be ~16% by 2050 [15]. The population over 80 years of age would become triple compared to the present number. Unprecedently, in the history of the human census, the aged population (≥65 years) globally had become more than the children (<5 years) in 2018 [15]. Hence, managing the aged population is a challenge for the healthcare sector.

Proper health maintenance remained an unaccomplished aspiration for the present aged population. Lack of vitality, diminished muscle strength, osteoporosis, joint pains, cardiovascular issues, urological problems, and cancers are preying this growing population worldwide. Unfortunately, the available remedies are limited. Moreover, side-effects and dependency on specific medication remain another concern. A solution has to be attained rapidly, proper supplementation of required vitamins, minerals, and other essential vitalizing factors could be provided to maintain the wellbeing of the aged population. Alternative therapies such as yoga, herbal medicine, and exercises proved to be effective in managing age-related issues.

The present 12-weeks study assessed the effect of Ashwagandha root extract supplementation on the possible improvement of the various domains of QoL and sleep parameters in the elderly population.

## Materials and methods

Initially, 74 participants were screened for participation and assessed for eligibility. Out of them, 50 participants met inclusion criteria and were enrolled to participate. All of them underwent randomization and were allocated respective medicinal packs of either treatment or placebo in a ratio of 1:1. The clinical study protocol followed in this study is provided in detail in the following section. 

Study design

A 12-week-long randomized, double-blind, placebo-controlled, prospective clinical study was designed to evaluate the efficacy and safety of the Ashwagandha root extract in boosting and maintaining the general well-being of the elderly people. Following the international mandate, the study protocol was designed as per the Declaration of Helsinki developed by the World Medical Association (2013 amendment). In addition, approval from the Institutional Ethics Committee (IEC) of Snehal Hospital, Naupada, Gokhale Road, Thane(W)-400602, Maharashtra, India was obtained for this study (Approval No: ECR/356/INST/MH/2013). The study was conducted in accordance with the Good Clinical Practice (GCP) guidelines, issued by the Indian Council of Medical Research (ICMR), Government of India. The entire study was conducted and reported following the Consolidated Standard of Reporting Trials (CONSORT) statement. 

Participant details

The present study population included 50 healthy elderly adults aged between 60 and 85 years. Study participants were selected from different outpatient clinics in the city of Thane. Participants were informed about the study details. Upon agreement, they were assessed by the principal investigator for eligibility, based on the predetermined inclusion and exclusion criteria. The ECOG (Eastern Cooperation Oncology Group) performance scale that describes the patient’s ability towards self-care, physical and daily activity statuses was used to assess the eligibility criteria [16].

Inclusion criteria

Elderly adults of either sex, aged between 60 and 85 years, were included in this study. Participants with an ECOG performance score of 0 to 1 were chosen for the study. Participants were also required to have a body mass index (BMI) value between 22 and 32 kg/m^2^ and body weight ≥ 50 kg. Participants who were willing to comply with the protocol and likely to be compliant with the prescribed product were included in the study.

Exclusion criteria

Participants with known renal insufficiency, having any history of renal failure at screening, were not considered. Those who were using hormone replacement therapy were also excluded from participation in this study. Moreover, participants with uncontrolled diabetes mellitus and hypertension were also excluded from the study. Existence of current or previous documented history of any chronic inflammatory condition including chronic infection like tuberculosis, leprosy, HIV, and collagen vascular diseases was also considered ineligible for participation. Individuals with known hemorrhagic problems, coagulation, or bleeding issues within 90 days prior to the screening were also excluded from the study.

Investigational products

Ashwagandha root extract and placebo were used for the treatment in capsulated form. Both types of capsules, i.e., Ashwagandha root extract intervention, and the placebo were weighing 300 mg and were produced in a Good Manufacturing Practice (GMP)-certified facility. All the capsules used in this study were identical in appearance, shape, color, and packaging. The placebo capsules contained starch as inert filler. Further, placebo capsules were kept with a cloth-covered envelope containing Ashwagandha root extract so that the odor of Ashwagandha permeates the placebo capsules. The investigational product KSM66 Ashwagandha root extract was received as a gift sample from its manufacturer, Ixoreal Biomed Inc., Los Angeles, California, USA.

Interventions

The treatment group received 300 mg of Ashwagandha root extract twice daily with water for 12 weeks in capsule form. The control or placebo group received an identical dose of placebo capsules. All the participants were evaluated at baseline and after four, eight, and 12 weeks with the outcome measures as described below. Data on safety and adverse effects were collected at the end of 12 weeks. Patient's Global Assessment of Tolerability to Therapy (PGATT) and Physician’s Global Assessment of Efficacy to Therapy (PGAET) were also determined at 12 weeks.

Study procedure 

Informed consent was obtained from the participants during screening and enrollment. The participants were screened for brief medical history, general physical examination, and vital parameters. Once enrolled, the participants were assessed for the efficacy parameters for quality of life (QoL) and sleep assessments which included mental alertness on rising and sleep quality. The safety parameters were assessed based on adverse event reporting.

Sample size

The study was exploratory, the total sample size decided was 50 where 25 participants were allotted in each arm (placebo and intervention). The determined sample size was not based on any distributional assumptions or statistical calculations. The sample size was kept equivalent to the earlier studies available in the literature.

Randomization and blinding

Following the screening, research coordinators randomized the eligible participants through computer-based predetermined randomization (Rando version 1.2) in a 1:1 ratio to receive either Ashwagandha-root extract or placebo. The randomization list had non-stratified blocks of the same length. The study was a double-blind one, i.e., doctors and participants were unaware of participants receiving the treatment and the placebo. The treatment and placebo medication packs were made tamper-proof and identical in appearance and weight. All the packs were coded to conceal their contents along with proper labeling that contained the subject serial number (ID of the study). After the participants were enrolled, they were provided with the medication pack having the corresponding serial number only. During data collection, the research coordinators, the study investigators, and the attending care personnel were not allowed to access the randomization codes and allocations. Unblinding was allowed only after completion of the entire data collection or in case of any serious adverse event. The randomization codes were covered in aluminum foil and placed in a separate sealed envelope for each patient. Data analysts and responsible personnel of reporting study results were also unaware of the identity of the study groups. The data were double-entered and blinded to the statisticians as well

Primary outcome through WHOQOL- BREF questionnaire

The participants’ quality of life (QoL) was assessed by using the WHOQOL-BREF scale which is an established measurement method, used and evaluated earlier [17]. WHOQOL-BREF scale contains four domains including physical health, psychological condition, social relationships and environmental factors with a total of 26 questions. Raw scores for each domain was calculated by summing up the values of single items, and the final score was normalized to a value ranging between 0 and 100, zero (0) being the lowest and 100 being the highest value. The mean score of each domain and the total score were calculated. This questionnaire was translated into the Hindi language considering the ease of understanding of the participants. Later on, the outcomes were translated back to English to assess the liability of the measurement scale.

Secondary Outcome Measures

Sleepiness Scale

Sleepiness Scale is a questionnaire containing 4 items that measure the likelihood of falling asleep on a scale of 0-3 during typical daytime activities. The four items assess four different situations such as sitting alone, sitting and talking to someone, lying down during daytime and watching TV/movie/show that is considered as part of the daily activities. The scoring is based on the probability of falling asleep during the four situations mentioned. The individual scores are summated to obtain a single number. The sleep scale score can range from 0-12. A higher score indicates excessive sleepiness. 

Mental Alertness on the Rise

After waking up every morning, mental alertness on rising was assessed on a three-point scale perceived by the patient as Alert, Slightly drowsy, and Extremely drowsy. The scores were assessed at baseline, week 4, week 8 and week 12 respectively. 

Sleep quality

Sleep quality was assessed using a seven-point Likert scale to estimate and analyze the overall sleep quality as perceived by the patient after waking up in the morning. The score representation is as follows: Excellent = 1, Very Good = 2, Good = 3, Fair = 4, Poor = 5, Very Poor = 6, and Extremely Poor =7. As evident from the scoring pattern, a higher score indicates poor quality of sleep. 

Safety assessment

Plausible adverse reporting and safety assessment was conducted for all the participants of this study. At week 4, week 8, and week 12, participants were instructed to list any adverse effects, symptoms or illness experienced during the study period. 

Clinical safety and tolerability were assessed based on the adverse events reported by the participants during the follow-up or the clinical evaluation. Patient’s Global Assessment of Tolerability to Therapy (PGATT) was considered by the physician to assess the participants conditions on a five-point rating scale represented as Excellent tolerability (no adverse effects, and patient able to tolerate the drug), Good tolerability (minimal side effects not interfering with patient’s daily activities), Moderate tolerability (some side effects and minimal interference in patient’s daily activities), Poor tolerability (significant side effects and significant interference in patient’s daily activities), and Worst tolerability (patient not able to tolerate the drug at all due to adverse effects). 

Physician’s Global Assessment of Efficacy to Therapy (PGAET) was also considered by the physician to estimate the efficacy on a five-point rating scale as follows: Excellent efficacy, Good efficacy, Moderate efficacy, Poor efficacy, and Worst efficacy. Adverse events were recorded, along with their severity, duration and relationship to study drug. 

Statistical analysis

All the participants enrolled were analyzed according to their randomized group, regardless of compliance with the treatment or any other deviation from protocol. All relevant statistical calculations were done using Stata (version: 13.1) software. The analyses were conducted in both intent-to-treat (ITT) and per-protocol (PP) datasets. The obtained analysis outcome of ranking data and scores are tabulated here as mean ± standard deviation (SD). To ensure the best statistical practices, 95% confidence intervals (CI) were accepted for the study. The two treatments were compared for differences using the unpaired t-test for continuous variables.

Multivariate analysis for the changes in the scores from the baseline to the end of the study was done. Analysis for the considered 5 domains and the total scores of WHOQOL-BREF in the two groups were done using the General linear model (GLM). The regression method was chosen due to its superiority and flexibility over linear regression. Unlike general regression, the GLM method considers categorical variables and non-normal residuals also. Estimated means were computed for the covariates. Non-parametric data (ranking data) for mental alertness and sleep quality were compared between the two treatments (placebo and Ashwagandha) using the Chi-square test (Spearman’s). Global assessments and discrete data were compared using the Chi-square test.

## Results

**Figure 1:**
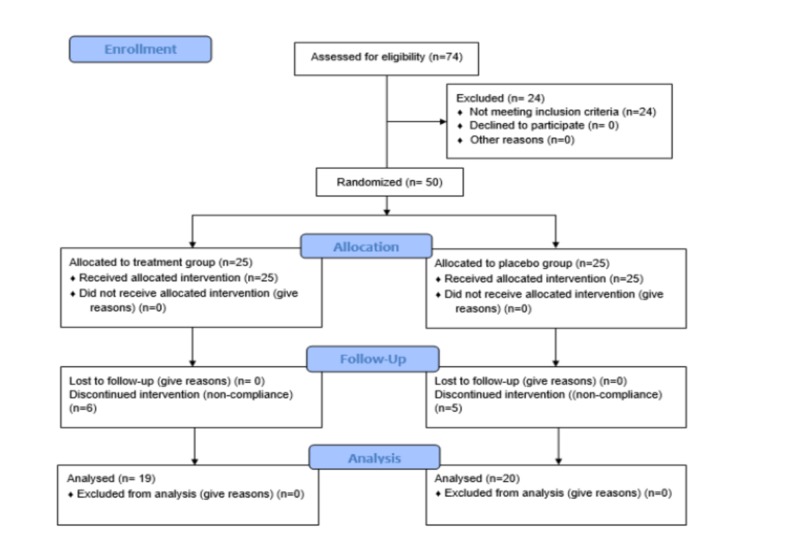
Representation of the CONSORT Flow diagram

The study was initiated with 50 participants, 25 in each arm. Later, as the study progressed, 39 participants complied with the necessary treatment requirements, i.e., consumed capsules, completed the self-reported questionnaires, etc. for 12 weeks. During the study course, six and five participants dropped out of the treatment and placebo group, respectively, due to noncompliance. No participant withdrew from the study due to self-reported adverse effects because of the capsule intake (Figure 1). The final per-protocol analysis was done using the data of the 39 participants.

Demographics

The mean age of participants was calculated using a per-protocol analysis of the 39 participants enrolled in the study. The mean age of the experimental and control group was documented as 72.16 ± 4.36 and 70.70 ± 4.47, respectively. The deviation among the participants’ age was less in both the groups; thus, we had homogeneous study participants of almost equivalent age groups. The demographic characteristics and baseline features of the two groups are represented in Table 1. The outcome indicates that the study population was quite homogenous with no significant statistical differences between the groups with reference to the baseline demographic characteristics. 

 

**Table 1:** Baseline demography and vital parameters in PP dataset (n=39) PP, per-protocol; BP, blood pressure; BMI, body mass index; CI, confidence interval.

	Treatment										
	Ashwagandha						Placebo				
	N	Mean	SD	95% CI			N	Mean	SD	95% CI	
				Lower		Upper				Lower	Upper
Age (yrs.)	19	72.16	4.36	63.61	80.71		20	70.7	4.47	61.94	79.46
Systolic BP (mm Hg)	19	153.95	12.33	129.78	178.12		20	152.1	7.29	137.81	166.39
Diastolic BP (mm Hg)	19	99.21	9.6	80.39	118.03		20	94.8	9.87	75.45	114.15
Heart Rate (per min.)	19	83.79	12.76	58.78	108.80		20	85.9	12.54	61.32	110.48
BMI (kg/sq.m)	19	26.25	4.28	17.86	34.64		20	27.55	3.92	19.87	35.23

Outcome measures

Primary Outcome of the WHOQOL-BREF Questionnaire

At baseline, the total mean score and standard deviation (SD) value of WHOQOL-BREF were found to be 140.53 ± 8.25, for the treatment group in the per-protocol dataset (n=19). On the other hand, the obtained mean value in the placebo group was 139.30 ± 9.65 in the per-protocol dataset (n=20) (Table 2). The global domain scores in both the treatment and placebo groups were having the least deviation among the domains scored. On the contrary, the environment domain resulted in a wide range of values in both groups. 

 

**Table 2:** WHOQOL-BREF questionnaire assessment outcome for the treatment and placebo group at baseline and end of therapy CI, confidence interval

		Ashwagandha						Placebo				
	N	Mean	SD	95% C.I.			N	Mean	SD	95% CI		
				Lower	Upper					Lower		Upper
Baseline												
Total Score	19	140.53	8.25	124.36		156.70	20	139.3	9.65	120.39	158.21	
Global domain	19	8.26	0.93	6.44		10.08	20	8.05	0.89	6.31	9.79	
Physical domain	19	36.11	4.81	26.68		45.54	20	36.9	3.04	30.94	42.86	
Psychological domain	19	32.05	3.36	25.46		38.64	20	31.15	2.62	26.01	36.29	
Social relationship domain	19	27.16	2.79	21.69		32.63	20	27	1.97	23.14	30.86	
Environment domain	19	36.95	5.02	27.11		46.79	20	36.2	4.4	27.58	44.82	
End of therapy (12 weeks)												
Total Score	19	161.84***	9.32	143.57		180.11	20	147.65	10.5	127.07	168.23	
Global domain	19	10.26 ***	0.93	8.44		12.08	20	9.05	0.89	7.31	10.79	
Physical domain	19	44.21***	3.01	38.31		50.11	20	39.75	3.35	33.18	46.32	
Psychological domain	19	38.11***	2.23	33.74		42.48	20	33.45	2.58	28.39	38.51	
Social relationship domain	19	28.68	2.14	24.49		32.87	20	28.2	2.07	24.14	32.26	
Environment domain	19	40.58 *	3.96	32.82		48.34	20	37.2	4.4	28.58	45.82	
* and *** indicates p < 0.05 and p < 0.0001 respectively for a two-tailed T-test for improvement from baseline being higher than the placebo.												

The mean score for all the domains including global domain score, physical, psychological, social relationship, and environmental was found to be improved in the treatment group at the end of the study. In parallel, the placebo group also showed a slight improvement in scores for all the domains but the changes were comparatively lower compared to the treatment group (Table 2).

Statistical assessment of the changes between the placebo and treatment group were assessed for each component of the WHO-BREF Quality of life questionnaire assessment outcomes. The results revealed that all the changes observed in individual domains such as total score (P<0.0001), global domain(P<0.0001), physical domain (P<0.0001), psychological domain (P<0.0001), and environmental domain (P<0.016) were found significant. The social relationship domain showed improvement both in the treatment and in the placebo group but was not found statistically significant (P=0.476). 

**Figure 2:**
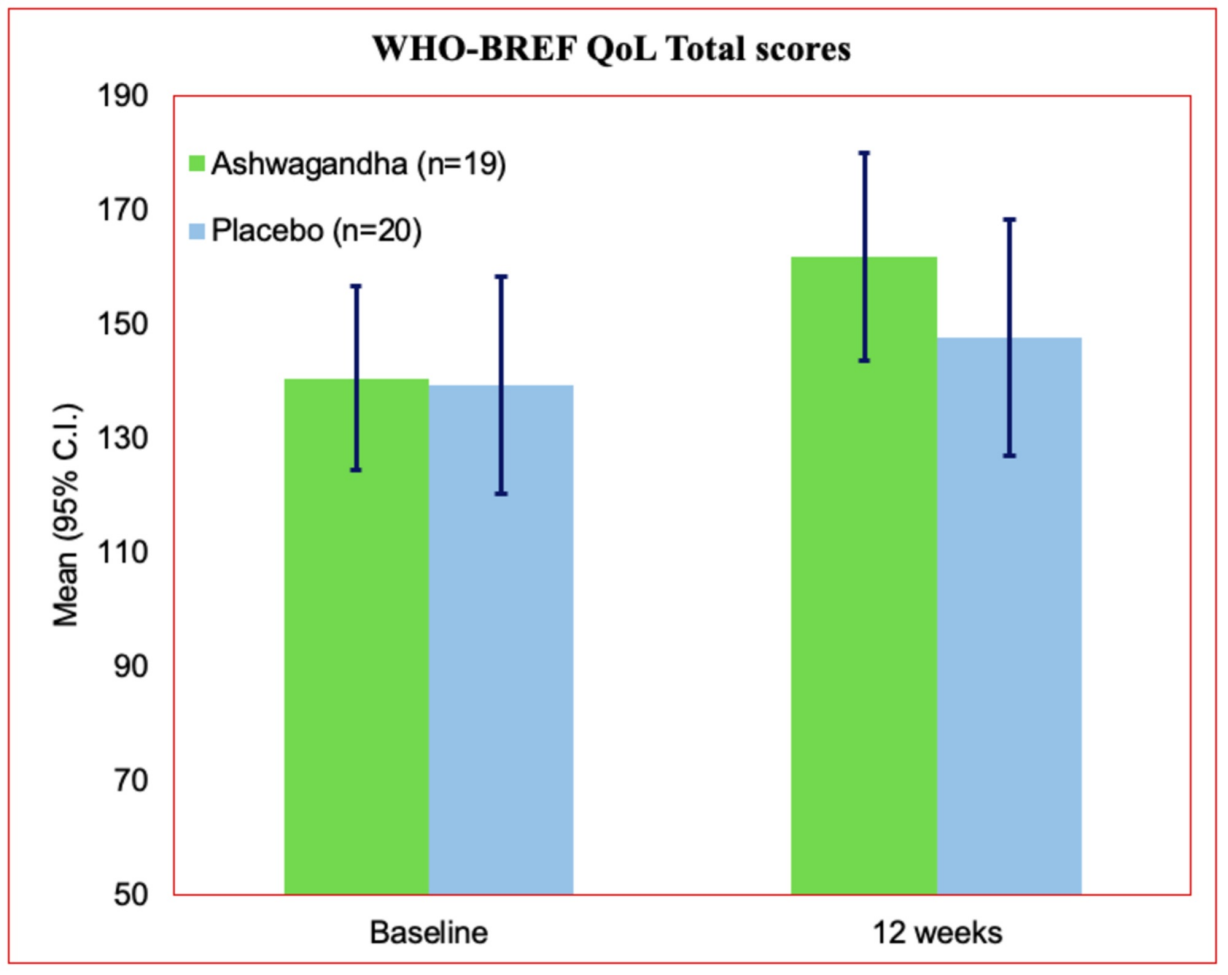
WHO-BREF Quality of life total scores

The comparative analysis of the total score obtained from the WHOQOL-BREF questionnaire assessment displayed a clear distinction and better outcome in the treatment group compared to their placebo counterpart in the present study as evident from Figure 2. 

Secondary outcome measures

For any elderly person, proper sleep is an important factor that determines their health condition and further health benefits. Moreover, complications with sleep, such as acute or chronic insomnia, in an elderly person can increase the risk for hypertension, cardiovascular diseases, urological complications, neurological conditions, and other unwanted health issues. Therefore, sleep parameters were given due importance and were considered in this study. 

Sleep scale

The baseline scores of the sleep scale were noted as 7.53 ±1.87 and 7.45 ±3.24 in the treatment and placebo group, respectively (Table 3). A gradual decrease in the sleep score was witnessed during this study from baseline to the end of the therapy. Screening at week 4, and week 8, demonstrated sleep scores as 6.79 ±1.84 and 6.37 ±1.83, respectively, for the treatment group. At the end of the study (week-12), the mean sleep score in the treatment group was noted as 5.00 ±1.89. 

**Table 3:** SS scores, mental alertness on rising, and sleep quality on a 7-point Likert scale in PP dataset (n=39) SS, sleep scale; PP per-protocol.

Sleep Scale scores	Ashwagandha			95% CI		Placebo			95% CI	
	N	Mean	SD	Lower	Upper	N	Mean	SD	Lower	Upper
Baseline	25	7.53	1.867	3.87	11.19	25	7.45	3.236	1.11	13.79
Week 4	19	6.79	1.843	3.18	10.40	20	7.1	3.076	1.07	13.13
Week 8	19	6.37	1.832	2.78	9.96	20	6.85	2.996	0.98	12.72
Week 12	19	5.00	1.886	1.30	8.70	20	6.35	2.996	0.48	12.22
Mental alertness on rising	Ashwagandha			95% C.I.		Placebo			95% C.I.	
	N	Mean	SD	Lower	Upper	N	Mean	SD	Lower	Upper
Baseline	25	2.24	0.663	0.94	3.54	25	2.2	0.645	0.94	3.46
Week 4	19	1.47	0.612	0.27	2.67	20	1.7	0.733	0.26	3.14
Week 8	19	1.16*	0.375	0.43	1.90	20	1.55	0.686	0.21	2.89
Week 12	19	1.05*	0.229	0.60	1.50	20	1.35	0.587	0.20	2.50
Sleep quality on 7-point Likert scale	Ashwagandha			95% C.I.		Placebo			95% C.I.	
	N	Mean	SD	Lower	Upper	N	Mean	SD	Lower	Upper
Baseline	25	5.76	0.663	4.46	7.06	25	5.72	0.737	4.28	7.16
Week 4	19	4.53	1.124	2.33	6.73	20	5.05	0.999	3.09	7.01
Week 8	19	3.47**	1.124	1.27	5.67	20	4.55	0.686	3.21	5.89
Week 12	19	2.47***	1.073	0.37	4.57	20	4.3	0.657	3.01	5.59
*, ** and *** indicates p < 0.05, p < 0.001 and p < 0.0001 respectively for Two-tailed T-test for improvement from baseline being higher than the placebo.										

**Figure 3:**
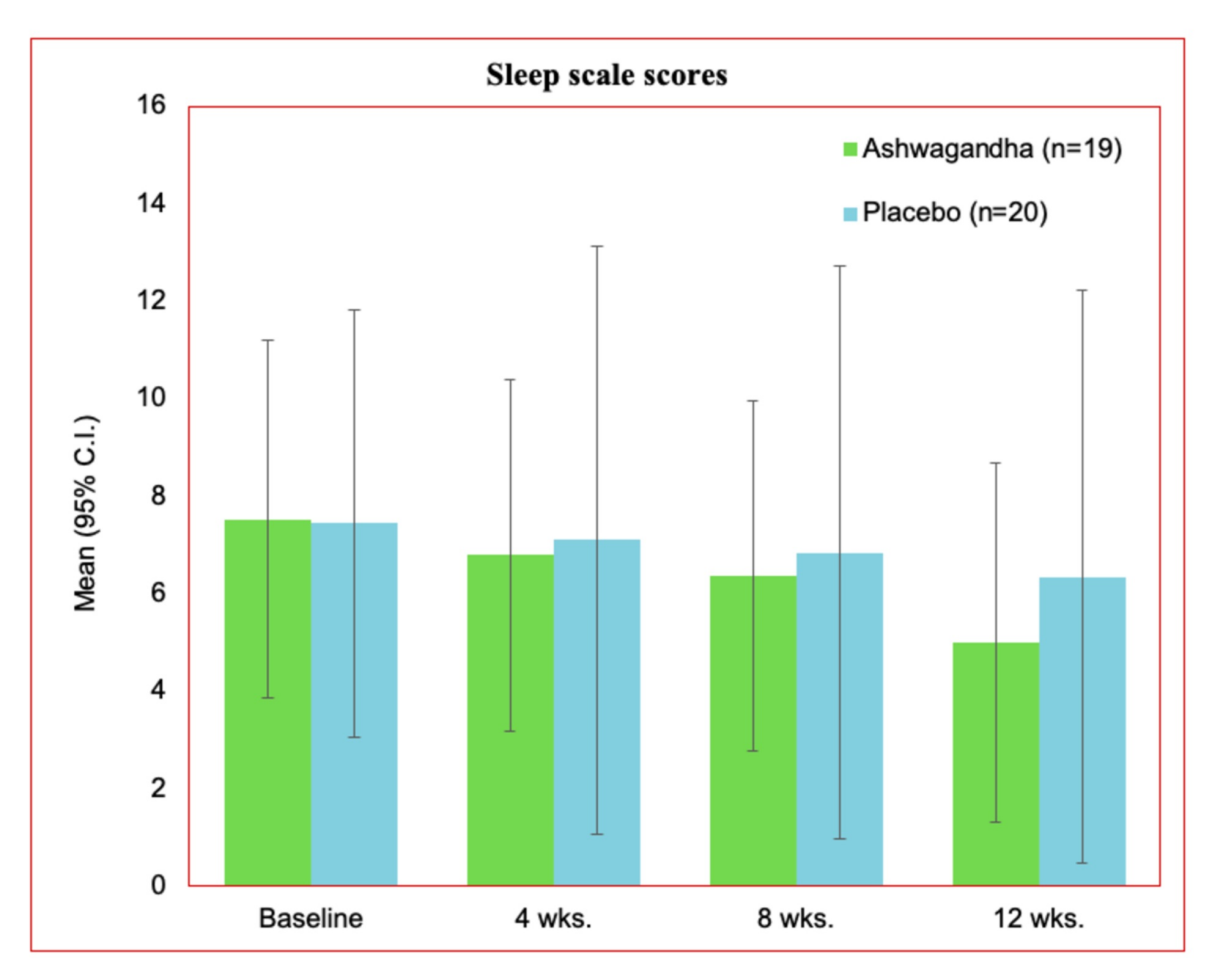
Sleep scale scores for treatment and placebo group for the per-protocol dataset C.I., confidence interval

A reduction in sleep score signifies better health status and sleep quality. The gradual changes in the mean sleep scale values are represented in Figure 3. The better impact of the Ashwagandha treatment was recorded compared to the placebo in this study. There was a significant decrease in the sleep scale scores at the end of the study from the observed baseline values.

Mental alertness on rising

Quality sleep outcome also impacts wakefulness. Proper sleep supports improved alertness and rejuvenated wakefulness after sleep which is vital for every human being. Thus, mental alertness on rising provide an indirect assessment of the quality of sleep. In the present study, mental alertness on rising was assessed on a 3-point scale. The obtained outcomes are presented in Table 3. An improved outcome was recorded for the Ashwagandha group (1.05 ± 0.23) compared to their placebo counterpart (1.35 ± 0.587). Lower mean outcome value represents better alertness and less drowsiness.

**Figure 4:**
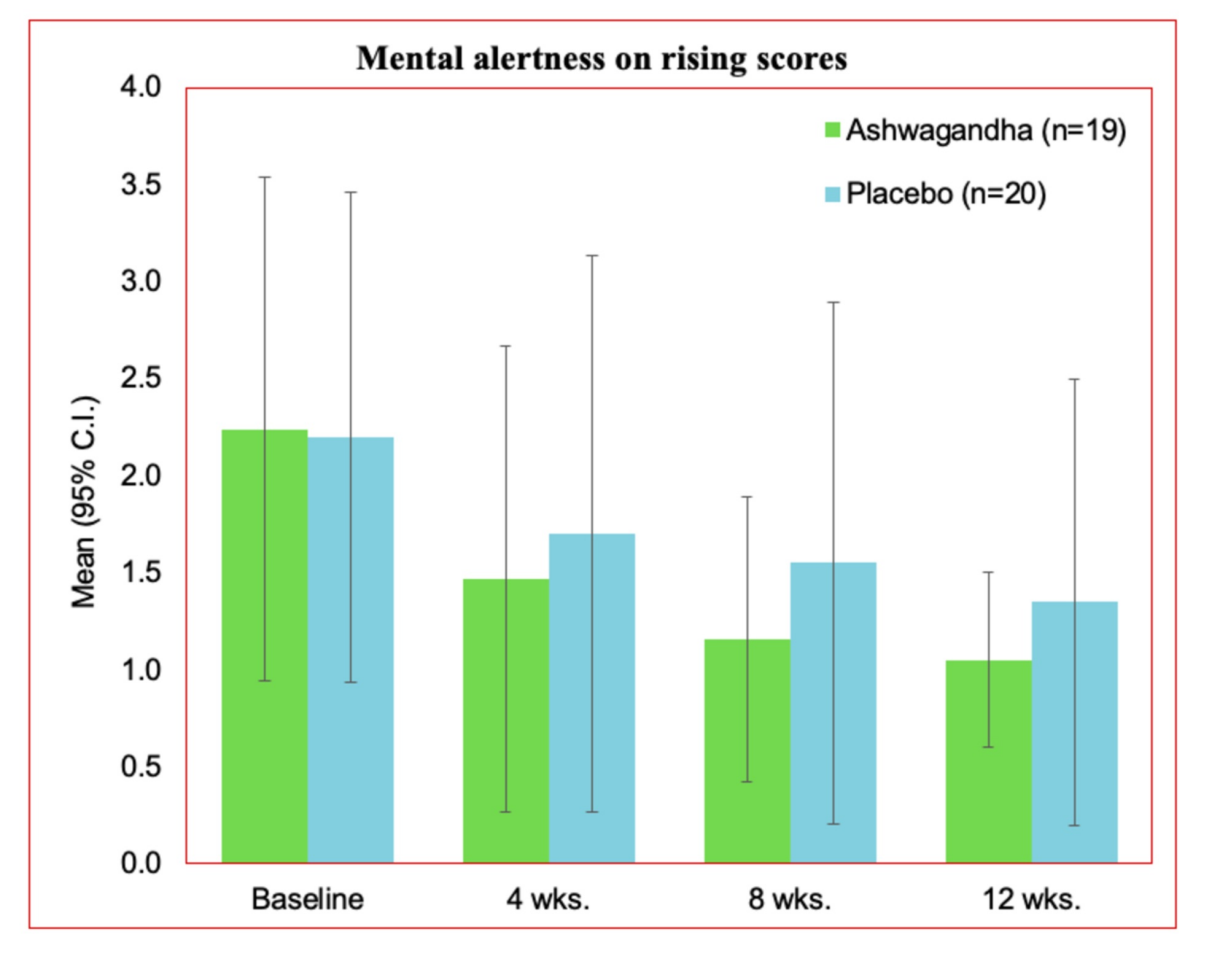
Mental alertness on rising at 12 weeks in the PP dataset

The comparative representation of the mental alertness on rising is presented in Figure 4. A significant increment in the alertness is witnessed at the end of the study (week 12) compared to the baseline estimations for the participants. 

Sleep quality

In parallel to sleep scale, sleep quality was also assessed for the participants in this study. The scale represents the quality in an inverse way, higher the score, lower the quality of sleep. On a scale of 7, the baseline sleep quality for both the groups (treatment and placebo) were recorded quite higher. At baseline, the mean and SD for the treatment group was 5.76 ± 0.66. For the placebo group, the mean and standard deviation values recorded were 5.72 ± 0.73. The quality of sleep was observed to be increasing gradually with decreasing score (Table 3) in every periodic assessment. The periodic assessment included the baseline, followed by week 4, week 8, and the end of the study (week 12). The final mean score for the sleep quality was 2.47 ± 1.07 in the treatment group, and 4.3 ± 0.65 in the placebo group. The observed changes between the Ashwagandha and the placebo group were found statistically significant at week 8 (P 0.001) and at week 12 (P<0.0001).

**Figure 5:**
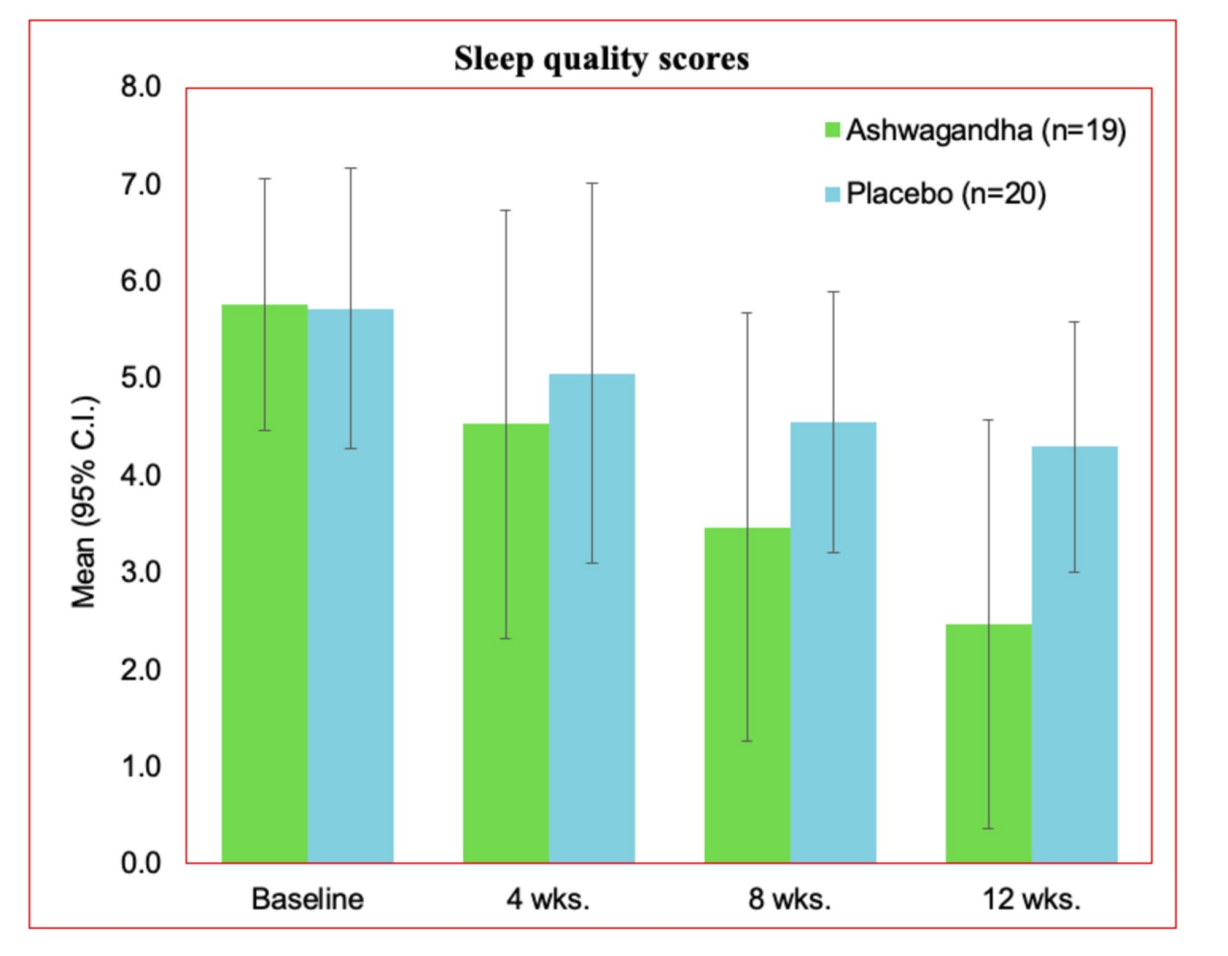
Sleep quality scores in the PP dataset

The comparative representation of the sleep quality scores obtained for the assessment periods considered is presented for both the groups (Figure 5). A significant difference was obtained in the outcome where a better sleep quality score was achieved in the treatment group.

Adverse reporting and safety assessment

Ashwagandha was reported well-tolerated among the participants and no significant report of adverse events was received from the placebo and treatment group members. The assessment and reporting of the possible adverse effect, safety measurement, efficacy assessment, tolerability estimation was conducted using PGAET and PGATT reporting system (Table 4).

The PGAET assessment suggests that in the treatment group, for most of the participants, the therapeutic material rendered either “Excellent” (14, 73.70%) or “Good” (5, 26.30%) outcome. For the placebo group, different outcomes were observed where “Satisfactory”(4, 20%) and “Poor” (4, 20%) efficacy was also mentioned (Table 4). 

The PGATT outcomes suggest that the participants reported either excellent (12 participants) or good (7 participants) tolerability of the therapy for the Ashwagandha root extract. A similar outcome was reported for the placebo. The test product was safe and tolerable among the participants and no adverse event was reported by any of the participants. Moreover, the self-assessment outcomes suggest that the study population had improved general well-being. 

**Table 4:** PGAET and PGATT assessment outcomes for the PP dataset PGAET, Physician’s Global Assessments of Efficacy of Therapy; PGATT, Patient’s Global Assessments of Tolerability to Therapy; PP, per-protocol

	Ashwagandha (n=19)		Placebo (n=20)	
PGAET	N	%	N	%
Excellent	14	73.70%	5	25.00%
Good	5	26.30%	7	35.00%
Satisfactory	0	0.00%	4	20.00%
Poor	0	0.00%	4	20.00%
	Ashwagandha (n=19)		Placebo (n=20)	
PGATT	N	%	N	%
Excellent	12	63.20%	17	85.00%
Good	7	36.80%	3	15.00%
Satisfactory	0	0.00%	0	0.00%
Poor	0	0.00%	0	0.00%

## Discussion

Aging is a prevalent and continuous biological process that is affected by physiological, social, psychological, and environmental factors. Aging is found associated with several disease conditions and characterized by a gradual functional decline posing a significant risk for neurodegenerative diseases, cancers, cardiovascular diseases, and diabetes [18]. Ayurveda considered aging as an important health context since ancient times [19]. It is referred to as “Jara” (aging or declining phase) in classical Ayurveda. 

In modern medicine, a great number of studies on animal models and human cell lines are ongoing pertaining to aging, senescence, and rejuvenation [20]. Reports suggest that aging is associated with a decline in sleep quality and altering sleep patterns [21]. A study with a large number of participants suggested that 42% of the older subjects were having problems in sleep initiation and maintenance [22]. Studies suggest that apart from the plateau maintained in the middle age, growing age and sleep time is having an inverse relation. Lighter sleep patterns, frequent arousal, and lack of deep sleep become prominent with aging. At the molecular level, such alteration in sleep pattern occurs due to reduced melatonin surge, presence of advanced sleep phase syndrome, lack of efficiency in internal circadian clock and circadian oscillation. Shorter telomere length also found to be associated with sleep deficiency in the elderly population.

Ashwagandha supplementation could be an effective alternative that can counter aging issues including sleep quality and mental alertness improvement. Earlier evidence suggests that Ashwagandha impacts positively on overall well-being including an antioxidant effect which is essential in age-related problems [23].

Several clinical studies have proven the efficacy, tolerability, and safety of Ashwagandha's use in various contexts. The root extract of Ashwagandha was found effective in enhancing cardiorespiratory endurance, improving muscle strength and recovery, and effective in general bodyweight management when compared to the respective placebo [24-26]. Apart from the contribution in general well-being, *W. somnifera* root extract also helps in improving sexual function in both men and women [6,27].

Ayurvedic medicine system accepts that Ashwagandha is having a vital role in improving longevity, but evidence in the light of modern research is limited. Kumar et al. reported that the purified root extract of Ashwagandha can increase the lifespan of *C.elegans* approximately 20% more than their normal lifespan [7]. Even though no such studies are available in humans so far, the study outcome may unveil a great possibility for the future. All these pieces of evidence are having great cues to understand the impact of Ashwagandha in general health and wellbeing.

As an excellent multipurpose natural supplement, it was an obvious choice to explore the benefits of Ashwagandha in the aging population. The present study was carried out to observe the impact of Ashwagandha supplementation in the elderly population in terms of safety, efficacy, tolerability, augmenting better sleep, and rejuvenation. Ancient texts and Ayurvedic treatment regimes had proven the benefits of Ashwagandha in many disease conditions, enriching vitality, and rejuvenation. Scientific reports suggested that Ashwagandha contains sleep-inducing compounds that aid in better sleep in all age groups. We reported a similar impact of Ashwagandha root extract on insomnia and anxiety recently [28]. Equivalent to modern geriatric medicine, “Jara Chikitsa” (Aging treatment) and “Jara Vyadhi” (Aging-associated diseases), remained an important part of the classical Ayurvedic treatment process where Ashwagandha is recommended as one of the most effective solutions [29]. Ashwagandha has a proven impact on improving memory and cognition for healthy beings and in mild cognitive impairment (MCI) [30]. Thus, the extract of Ashwagandha is having the potential to counter most of the prominent aging-related issues.

The outcome of the present study suggests significant improvement of sleep condition, mental alertness, and quality of life (QoL) in elderly participants who received Ashwagandha root extract in comparison to those who took a placebo. Therefore, Ashwagandha root extract could be an acceptable and admirable alternative supplement in improving various age-related health issues and may boost overall general wellbeing in an elderly person. Further exploration with larger and diverse elderly patient populations could shed more insight into establishing and generalizing the results.

Limitations

The present study was conducted in a single study site and with smaller sample size. A multi-centered study with a longer duration and large subject groups, having diverse backgrounds, may provide more information on the various biochemical, physiological and psychological aspects and would also give a better understanding of the long-term effects of Ashwagandha in elderly people.

## Conclusions

The present study was conducted to assess the safety, tolerability, and efficacy of Ashwagandha supplementation for the aged population. It was observed that the supplement was well tolerated and safe for the study population. Moreover, the test product was helpful in managing general wellbeing, sleep, and mental alertness on rising, in the study population.
